# *Trichuris* WAP and CAP proteins: Potential whipworm vaccine candidates?

**DOI:** 10.1371/journal.pntd.0010933

**Published:** 2022-12-22

**Authors:** Eleanor Wainwright, Rebecca K. Shears

**Affiliations:** 1 School of Biological Sciences, Faculty of Biology, Medicine and Health, Manchester Academic Health Science Centre, University of Manchester, Manchester, United Kingdom; 2 Centre for Bioscience, Manchester Metropolitan University, Manchester, United Kingdom; 3 Department of Life Sciences, Faculty of Science and Engineering, Manchester Metropolitan University, Manchester, United Kingdom; Washington University in St Louis School of Medicine, UNITED STATES

## Abstract

*Trichuris trichiura* and *T*. *suis* are gastrointestinal dwelling roundworms that infect humans and pigs, respectively. Heavy infections cause gastrointestinal symptoms and impaired growth and development. Vaccination has the potential to reduce the disease burden of whipworm infection; however, there are currently no commercially available vaccines against these parasites and very few against other gastrointestinal-dwelling nematodes of medical and agricultural importance. The naturally occurring mouse whipworm, *T*. *muris*, has been used for decades to model human trichuriasis, and the immunogenic potential of the excretory/secretory material (E/S, which can be collected following ex vivo culture of worms) has been studied in the context of vaccine candidate identification. Despite this, researchers are yet to progress an effective vaccine candidate to clinical trials. The *T*. *muris*, *T*. *trichiura*, and *T*. *suis* genomes each encode between 10 and 27 whey acidic protein (WAP) domain-containing proteins and 15 to 34 cysteine-rich secretory protein/antigen 5/pathogenesis related-1 (CAP) family members. WAP and CAP proteins have been postulated to play key roles in host–parasite interactions and may possess immunomodulatory functions. In addition, both protein families have been explored in the context of helminth vaccines. Here, we use phylogenetic and functional analysis to investigate the evolutionary relationship between WAP and CAP proteins encoded by *T*. *muris*, *T*. *trichiura*, and *T*. *suis*. We highlight several WAP and CAP proteins that warrant further study to understand their biological function and as possible vaccine candidates against *T*. *trichiura* and/or *T*. *suis*, based on the close evolutionary relationship with WAP or CAP proteins identified within *T*. *muris* E/S products.

## Introduction

*Trichuris* is a genus of parasitic roundworms that infect the large intestine of their hosts. They are also known as whipworms due to their whip-like structure, with a thin anterior end that burrows into the epithelium and a thick posterior that is free to move about in the lumen [1]. There are over 70 species within the *Trichuris* genus, each with a different host, including *T*. *trichiura* (humans), *T*. *suis* (pigs), and *T*. *muris* (mouse). Genome and transcriptome analysis of these 3 species was completed in 2014 [2,3]. *T*. *trichiura* is the causative agent of human trichuriasis, a neglected tropical disease that affects 477 million people worldwide, causing impaired growth and physical development of children, and gastrointestinal symptoms including diarrhoea, abdominal pain, and rectal prolapse in individuals with heavy worm burdens [1,4]. *T*. *suis* causes similar symptoms in pigs, and thus is a burden for the agricultural industry. A combination of better sanitation, anthelminthic drugs, and protective vaccines are predicted to reduce the morbidity caused by *Trichuris* species [4,5]. Alarmingly, recent evidence suggests that the currently available anthelminthic drugs have limited efficacy against *T*. *trichiura* and *T*. *suis*, and that resistance may be arising in endemic areas with intense treatment regimes. There is also a high rate of posttreatment reinfection, emphasising the need for prophylactic vaccines against these parasites [6–8]. Progress towards vaccines to protect against whipworm infections has been hindered by a lack of known protective antigens and limited information on the biology of these parasites, despite decades of research involving the closely related, naturally occurring mouse whipworm, *T*. *muris* [2,9,10].

The *T*. *muris* mouse model has been critical for enabling researchers to understand how *Trichuris* species interact with their host. The immune response to *T*. *muris* is dependent on the dose—a low-dose infection (20 to 25 eggs) induces a Th1 polarised response and results in chronic infection, whereas a high-dose infection (200 eggs) results in Th2 immunity and worm expulsion [11]. *T*. *muris* secretes a myriad of proteins, RNAs, lipids, and extracellular vesicles (EVs) that can be collected following ex vivo culture of worms [9]. These excretory/secretory (E/S) products have formed the basis of vaccine design thus far, with researchers showing that vaccination with E/S induces protective immunity, enabling expulsion of low-dose infections, which ordinarily result in chronicity [12,13]. A variety of proteins have been explored as vaccine candidates using the *T*. *muris* mouse model, including a whey acidic protein (WAP), referred to as *Tm-*WAP49, a chymotrypsin-like serine protease and 2 chitin-binding domain-containing proteins [14–16]. However, none of the candidates explored to date have induced particularly effective protective immunity (protection is partial against a high-dose infection only), aside from the major E/S protein, p43 [17], although the protective immunity observed with native p43 has yet to be recapitulated with recombinant p43 (Allison Bancroft, personal communication).

## Phylogenetic and functional analysis of *Trichuris* WAP and CAP proteins

*T*. *muris* E/S contains at least 13 WAP proteins and 10 CAP proteins (listed in Tables 1 and 2, respectively) [13,18]. It is our belief that these proteins may warrant further study as vaccine candidates for *Trichuris* species, given the large number of family members encoded within the genomes of *T*. *muris*, *T*. *trichiura*, and *T*. *suis* (25, 20, and 10 WAP and 34, 19, and 14 CAP proteins, respectively) (WormBase ParaSite) [19], as well as their possible roles in host–parasite interactions and immunomodulatory properties [2,20–25].

**Table 1:** List of WAP proteins identified within *T*. *muris* E/S.

UniProt ID	Species	Reference	Notes	Accession code
Not available	*T*. *muris*	[13,18]	This is the WAP protein investigated by Briggs and colleagues (2018) and was identified in both E/S and EVs, however the accession code is not recognised by the new version of WormBase ParaSite.	TMUE_0165000300
A0A5S6QNB6	*T*. *muris*	[13]	Also identified in EVs by Eichenberger and colleagues (2018).	TMUE_2000008851
A0A5S6QGR6	*T*. *muris*	[13]	Also identified in EVs by Eichenberger and colleagues (2018)	TMUE_2000006596
A0A5S6QK39	*T*. *muris*	[13]		TMUE_2000007534
A0A5S6QHN5	*T*. *muris*	[13]	Also identified in EVs by Eichenberger and colleagues (2018)	TMUE_2000006703
A0A5S6QHV5	*T*. *muris*	[13]		TMUE_2000006725
A0A5S6QPC9	*T*. *muris*	[13]	Also identified in EVs by Eichenberger and colleagues (2018)	TMUE_2000009080
A0A5S6PYW1	*T*. *muris*	[13]		TMUE_0000000156
A0A5S6QGL3	*T*. *muris*	[13]		TMUE_2000006541
A0A5S6QXY8	*T*. *muris*	[13]		TMUE_3000012251
A0A5S6QP59	*T*. *muris*	[18]	Identified in E/S and EVs by Eichenberger and colleagues (2018)	TMUE_2000008968
A0A5S6QIH3	*T*. *muris*	[18]	Identified in E/S and EVs by Eichenberger and colleagues (2018)	TMUE_2000006662
A0A5S6QNV5	*T*. *muris*	[18]	Identified in EVs only	TMUE_2000008844
A0A5S6QQ56	*T*. *muris*	[18]	Identified in EVs only	TMUE_2000009471

The WormBase accession code and reference(s) for the study in which each protein was identified are included. Those that were identified in EVs as well as soluble E/S components are noted in the “Notes” column.

**Table 2:** CAP proteins identified in *T*. *muris* and *T*. *suis* E/S.

UniProt ID	Species	Reference	Notes	Accession code
A0A5S6QWJ2	*T*. *muris*	[13,18]	Also identified in *T*. *muris* EVs (Shears and colleagues (2018b) and Eichenberger and colleagues (2018))	TMUE_3000011469 and TMUE_s0033006400
A0A5S6QW63	*T*. *muris*	[13,18]	Also identified in *T*. *muris* EVs (Shears and colleagues (2018b))	TMUE_3000011500
A0A5S6QWE3	*T*. *muris*	[13]	Identified in EVs (not soluble E/S component) by Eichenberger and colleagues (2018)	TMUE_3000011570
A0A5S6QGH1	*T*. *muris*	[13]		TMUE_2000006289
A0A5S6PZD5	*T*. *muris*	[13]		TMUE_2000010044
A0A5S6QVG5	*T*. *muris*	[13,18]	Also identified in *T*. *muris* EVs (Shears and colleagues (2018b) and Eichenberger and colleagues (2018))	TMUE_3000011103
A0A5S6QY82	*T*. *muris*	[13]		TMUE_3000012215
A0A5S6R3F2	*T*. *muris*	[18]	Identified in E/S and EVs by Eichenberger and colleagues (2018)	TMUE_3000013702
A0A5S6QQA1	*T*. *muris*	[18]	Identified in EVs (not soluble E/S component) by Eichenberger and colleagues (2018)	TMUE_2000009410
A0A5S6QW38	*T*. *muris*	[18]	Identified in EVs (not soluble E/S component) by Eichenberger and colleagues (2018)	TMUE_3000011470

The WormBase accession code and reference(s) for the study in which each protein was identified are included. Those that were identified in EVs as well as soluble E/S components are noted in the “Notes” column.

**Fig 1:**
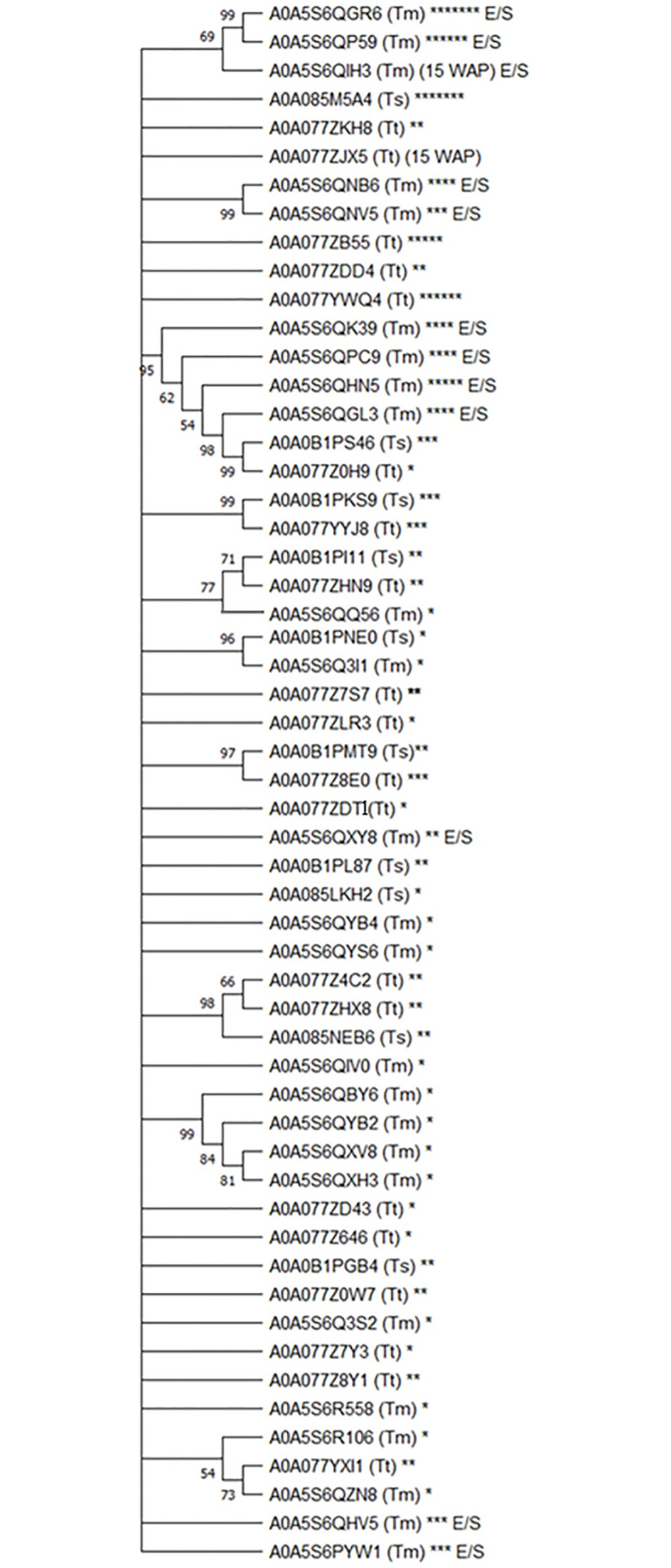
Phylogenetic tree denoting evolutionary relationship between *T*. *muris*, *T*. *trichiura*, and *T*. *suis* WAP proteins. The evolutionary history was inferred by using the maximum likelihood method and Whelan and Goldman + Freq. model. The bootstrap consensus tree inferred from 100 replicates is taken to represent the evolutionary history of the taxa analysed. Branches corresponding to partitions reproduced in less than 50% bootstrap replicates are collapsed. The percentage of replicate trees in which the associated taxa clustered together in the bootstrap test (100 replicates) is shown next to the branches. Initial tree(s) for the heuristic search were obtained automatically by applying Neighbor-Join and BioNJ algorithms to a matrix of pairwise distances estimated using the JTT model, and then selecting the topology with superior log likelihood value. This analysis involved 55 amino acid sequences. There were a total of 2,583 positions in the final data set. Evolutionary analyses were conducted in MEGA11. The number of asterisks denotes the number of WAP domains identified in each protein. “E/S” indicates proteins identified in *T*. *muris* E/S. Species is indicated in brackets (Tm = *T*. *muris*, Tt = *T*. *trichiura*, Ts = *T*. *suis*).

We carried out phylogenetic analysis to assess the evolutionary relationship between WAP proteins across *T*. *muris*, *T*. *trichiura*, and *T*. *suis* and coupled this to functional analysis (using InterPro software) to predict the number of WAP domains for each protein (Fig 1). We have also indicated which of these proteins have been previously identified in *T*. *muris* E/S (further information can be found in Table 1). Two proteins identified in *T*. *suis* E/S (S1 Table) that had originally been described as WAP proteins [26] did not appear to possess a WAP domain based on our analysis using InterPro. As such, these proteins were not included in Fig 1. Unfortunately, we were unable to include the vaccine candidate, *Tm-*WAP-49 in these analyses, as the sequence data is no longer available on WormBase (gene failed to map onto the new version of the genome assembly) [19].

The majority of *Trichuris* WAP proteins have 1–3 WAP domains, with some exceptions, including several *T*. *muris* WAP proteins containing 4–7 WAP domains, all of which have been identified within E/S. Four of these proteins cluster together on the phylogenetic tree (Fig 1), suggesting that they may be closely related to each other. A0A0B1PS46 (3 WAP domains, *T*. *suis*) and A0A077Z0H9 (1 WAP domain, *T*. *trichiura*) also appear to be closely related to this group of *T*. *muris* WAP proteins. Incidentally, they share 71.63% homology (95% sequence coverage) with each other, although their nearest *T*. *muris* homolog is Tm16 [27]. Two proteins, A0A5S6QIH3 (*T*. *muris* WAP protein identified in E/S) and A0A077ZJX5 (*T*. *trichiura*) had 15 WAP domains; however, these do not cluster particularly close together on the phylogenetic tree (Fig 1), suggesting that they are not necessarily that closely related in evolutionary terms. Indeed, sequence alignment data suggests only 36.7% homology between these proteins (97% coverage).

From these data, there is no clear lead vaccine candidate; however, the 4 *T*. *muris* proteins that have been detected within parasite secretions along with the 2 closely related *T*. *trichiura* and *T*. *suis* proteins (A0A077Z0H9 and A0A0B1PS46, respectively) may warrant further study to evaluate their function(s) during infection and their potential as vaccine candidates. Detection of these WAP proteins within *T*. *muris* E/S suggests that these gene products are released in vivo and may contribute to the protective properties of E/S (as evidenced by previous vaccination studies) [12,13]. The presence of homologs in *T*. *trichiura* and *T*. *suis* is an important factor to ensure translation from preclinical vaccine studies using the *T*. *muris* mouse model to efficacy against *T*. *trichiura* and *T*. *suis*. In the absence of more detailed functional information, these factors may be good selection criteria to prioritise these proteins for further functional analysis and consideration as vaccine candidates.

**Fig 2:**
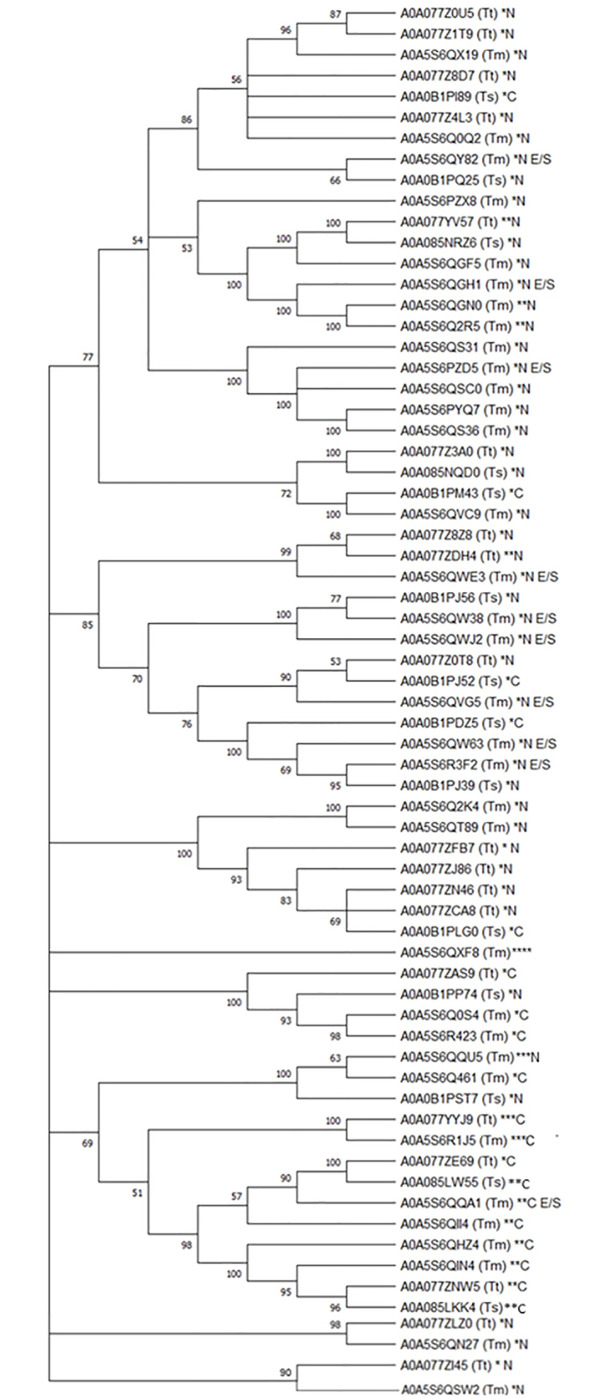
Phylogenetic tree denoting evolutionary relationship between *Trichuris* CAP proteins. The evolutionary history was inferred by using the maximum likelihood method and Whelan and Goldman + Freq. model. The bootstrap consensus tree inferred from 100 replicates is taken to represent the evolutionary history of the taxa analysed. Branches corresponding to partitions reproduced in less than 50% bootstrap replicates are collapsed. The percentage of replicate trees in which the associated taxa clustered together in the bootstrap test (100 replicates) is shown next to the branches. Initial tree(s) for the heuristic search were obtained automatically by applying Neighbor-Join and BioNJ algorithms to a matrix of pairwise distances estimated using the JTT model, and then selecting the topology with superior log likelihood value. This analysis involved 67 amino acid sequences. There were a total of 4,664 positions in the final data set. Evolutionary analyses were conducted in MEGA11. The number of asterisks denotes the number of CAP domains (1–3) identified in each protein. Whether these CAP domains are found at the N or C terminus of the protein is also indicated (N or C, respectively). “E/S” indicates proteins identified in *T*. *muris* or *T*. *suis* E/S. Species is indicated in brackets (Tm = *T*. *muris*, Tt = *T*. *trichiura*, Ts = *T*. *suis*).

Phylogenetic analysis was also carried out for *T*. *muris*, *T*. *trichiura*, and *T*. *suis* CAP proteins to assess the evolutionary relationship between these proteins. Functional analysis (InterPro) was also used to predict the number and location (N or C terminus) of CAP domains for each protein (Fig 2). Forty-eight of the 68 CAP family members identified across the 3 *Trichuris* species have N terminal CAP domains, while 19 members have C terminal CAP domains and 1 member had multiple CAP domains spanning the entire protein length (Fig 2). In fact, several members had multiple CAP domains, including 4 proteins (2 *T*. *muris* and 2 *T*. *trichiura)* with 2 to 3 CAP domain at the N terminus and 9 proteins with 2 to 3 CAP domains at C terminus. Ten family members were previously identified within *T*. *muris* E/S and/or EVs; however, 1 *T*. *muris* and 8 *T*. *suis* proteins (S1 Table) that had originally been described as CAP family members [13,18,25] did not appear to possess a CAP domain from our analysis using InterPro. As such, these proteins were not included in Fig 2.

CAP proteins of particular interest include A0A5S6QQA1, which was identified in *T*. *muris* E/S. This protein has a double CAP domain at the C terminus and is closely related to A0A085LW55 (*T*. *suis*) and A0A077ZE69 (*T*. *trichiura*). A0A085LW55 has double C terminal CAP domain; however, A0A077ZE69 has a single CAP domain. A0A085LW55 shares 52.3% (99% sequence coverage) with A0A5S6QQA1, while A0A077ZE69, which is roughly one-third of the length of A0A5S6QQA1 and A0A077ZE69 shares 63% homology with A0A077ZE69. A0A5S6QWE3 was also identified in *T*. *muris* E/S and has 2 closely related *T*. *trichiura* homologues: A0A077ZDH4 (49.5% homology, 99% sequence coverage) and A0A077Z8Z8 (57.5% homology, 85% sequence coverage). These *T*. *muris* proteins, which have been identified in E/S, with closely related homologs in *T*. *trichiura* and *T*. *suis* may be worth prioritising as vaccine candidates and/or for further functional testing.

## Discussion

Here, we carried out phylogenetic analysis on the WAP and CAP proteins identified from the *T*. *muris*, *T*. *trichiura*, and *T*. *suis* and genomes. We used the functional analysis tool, InterPro, to identify the number of WAP and CAP domains predicted for each protein, based on the predicted amino acid sequence, and highlighted which proteins have been previously identified within the secretions of *T*. *muris* and *T*. *suis*. Based on close evolutionary relationship to proteins identified by mass spectrometry in worm secretions, we highlight several WAP and CAP proteins that perhaps warrant further study as *T*. *trichiura* and *T*. *suis* vaccine candidates. Our rationale for this selection process is based on knowledge that vaccination of mice with *T*. *muris* E/S protects against subsequent infection, and thus WAP and CAP proteins that have been identified in these secretions may be more likely to have immunogenic properties. Of course, it will be important to determine the function of any vaccine candidates, as relatively little is known about the specific function of WAP and CAP proteins in *Trichuris* infection, despite the large number of gene products containing WAP and CAP domains.

The WAP domain gets its name from the WAP, a component of mammalian milk, and comprises 8 cysteine residues involved in forming 4 disulphide bonds [28]. A previous report stated that *T*. *muris* and *T*. *trichiura* genomes encoded several proteins with 1–9 WAP domains [2]; however, our analyses suggest that 1 *T*. *muris* and 1 *T*. *trichiura* protein contain 15 WAP domains, although the majority of *Trichuris* WAP family members contained 1–3 WAP domains. Mammalian WAP proteins have been shown to play diverse roles relating to the modulation of mucosal immunity, including inhibition of protease function, modulation of inflammation, wound healing, and antimicrobial activity [20]. It is possible that the WAP domain may impart 1 or more of these immunomodulatory functions to *Trichuris* WAP proteins; if this is the case, then inhibiting these functions through vaccination may represent a successful strategy for preventing/controlling whipworm infection in humans and other animals.

The CAP domain is 15 kDa in size and found in diverse organisms, including mammals and plants, where the domain is often referred to as the sperm coat protein (SCP) or pathogenesis related (PR) domain, respectively [21]. Relatively little is known about the role of CAP proteins in parasitic nematodes; however, there are multiple CAP family members within the secretomes of other helminths, including the well-studied rodent hookworms *Heligosomoides polygyrus* and *Nippostrongylus brasiliensis*, which have 25 and 37 CAP proteins, respectively (these are referred to as venom allergen-like (VAL) proteins in *H*. *polygyrus*) [21,22]. Our data suggest that the genomes of *T*. *muris*, *T*. *trichiura*, and *T*. *suis* each encode 15 to 34 CAP proteins. The historical hookworm vaccine candidates, *Ancylostoma*-secreted protein (ASP) and *Necator americanus (Na) Na-*ASP-1 and 2, are also members of CAP superfamily [23–25]. It was speculated that these proteins play an important role in establishing infection within the host and modulating the immune response, which was why they were investigated by vaccinologists looking to recapitulate the effects of vaccination with live attenuated larvae. *Na-*ASP-2 was progressed to Phase I clinical trials; however, vaccination of volunteers in Brazil (where *N*. *americanus* is endemic) resulted in generalised urticarial reactions in several volunteers, thought to be associated with preexisting *Na*-ASP-2-specific IgE induced by previous hookworm infection [29]. The allergenic potential of any future CAP protein vaccine candidates should be carefully monitored, ideally during preclinical studies (for example, by performing vaccination experiments in pre-infected animals and monitoring for any allergenic effects, as well as assaying for candidate-specific antibody responses in these pre-infected individuals). Despite the lack of information on the potential biological function of CAP proteins and concerns regarding allergenic potential, the CAP superfamily may warrant further exploration in relation to novel vaccine candidates for *Trichuris* species given that it is one of the most prominent protein families in terms of number of CAP proteins found in the secretory products and encoded in the genomes of these species.

In summary, given the large number of WAP and CAP proteins encoded in the genomes (and identified within secretions) of *Trichuris* species of scientific/medical/agricultural importance, as well as their possible roles in host–parasite interactions, we believe that these 2 classes of proteins warrant further study to assess their potential as whipworm vaccine candidates. Here, we highlight several proteins of interest, although we recommend that functional assays are performed to determine the biological role of candidates during infection, and that potential allergenic responses to candidates (particularly CAP proteins) are monitored during preclinical testing.

## Methods

### Identification of *Trichuris* WAP and CAP proteins

*T*. *muris*, *T*. *trichiura*, *and T*. *suis* WAP and CAP proteins were identified by searching for “WAP” and “CAP” using WormBase Parasite (https://parasite.wormbase.org/index.html) [19] and selecting the appropriate species. The predicted amino acid sequence was used to build phylogenetic trees.

### Building of phylogenetic trees

Amino acid sequences were aligned in MEGA11 software using MUltiple Sequence Comparison by Log-Expectation (MUSCLE) alignment. The resulting alignment was exported in MEGA format. The data modelling function was used to identify the most appropriate maximum likelihood model that the data fits. The model with the lowest Bayesian information criterion (BIC) value was selected, and the model settings were used to construct the phylogenetic tree. This was exported in Newick format and information on the number of WAP/CAP domains and identification of those proteins previously identified within secretory products was added.

### Functional analysis of WAP and CAP proteins

InterPro (EMBL-EBI) was used to identify the number and position of WAP and CAP (or ShKT) domains for each protein. A literature search was conducted to highlight those WAP and CAP proteins that have been previously identified in *T*. *muris* and *T*. *suis* E/S.

**Learning points**

- WAP and CAP proteins are numerous within *Trichuris* genomes; however, the role of these proteins in the context of whipworm infection is largely unknown.
- Both WAP and CAP family members have been explored as helminth vaccine candidates. *Tm-*WAP-49, a recombinant *T*. *muris* WAP protein, showed some efficacy in murine vaccination studies, while CAP family members, such as *Na-*ASP-1 and 2, have been explored as hookworm vaccine candidates.
- We highlight several WAP and CAP proteins that may warrant further study, based our phylogenetic analysis and/or identification of proteins or closely related homologs in *T*. *muris* secretions. These include 4 *T*. *muris* WAP proteins previously detected within parasite secretions along with the 2 closely related *T*. *trichiura* and *T*. *suis* proteins (A0A077Z0H9 and A0A0B1PS46, respectively) and A0A5S6QQA1, a *T*. *muris* CAP protein that was identified in E/S, along with its *T*. *trichiura* and *T*. *suis* homologs, A0A077ZE69 and A0A085LW55.
- WAP and CAP family members may warrant further study as whipworm vaccine candidates given the prominence of these proteins within *Trichuris* genomes and secretions, although potential allergenic responses to candidates should be assessed in preclinical animal models and functional assays should be performed to determine the biological role of candidates during infection.

**Key papers in the field**

- Foth BJ, Tsai IJ, Reid AJ, Bancroft AJ, Nichol S, Tracey A, et al. Whipworm genome and dual-species transcriptome analyses provide molecular insights into an intimate host-parasite interaction. Nat Genet. 2014 Jul;46(7):693–700. doi: 10.1038/ng.3010. Epub 2014 Jun 15. PMID: 24929830; PMCID: PMC5012510.
- Jex AR, Nejsum P, Schwarz EM, Hu L, Young ND, Hall RS, et al. Genome and transcriptome of the porcine whipworm Trichuris suis. Nat Genet. 2014 Jul;46(7):701–6. doi: 10.1038/ng.3012. Epub 2014 Jun 15. PMID: 24929829; PMCID: PMC4105696.
- Leroux LP, Nasr M, Valanparambil R, Tam M, Rosa BA, Siciliani E, et al. Analysis of the Trichuris suis excretory/secretory proteins as a function of life cycle stage and their immunomodulatory properties. Sci Rep. 2018 Oct 29;8(1):15921. doi: 10.1038/s41598-018-34174-4. PMID: 30374177; PMCID: PMC6206011.
- Eichenberger RM, Talukder MH, Field MA, Wangchuk P, Giacomin P, Loukas A, et al. Characterization of *Trichuris muris* secreted proteins and extracellular vesicles provides new insights into host-parasite communication. J Extracell Vesicles. 2018 Jan 21;7(1):1428004. doi: 10.1080/20013078.2018.1428004. PMID: 29410780; PMCID: PMC5795766.

**Advantages and disadvantages of the approaches used**

- We constructed 2 phylogenetic trees for *T*. *trichiura*, *T*. *muris*, and *T*. *suis* WAP and CAP family members (1 tree for each family) using MUSCLE alignment in MEGA11 (amino acid sequences are available on WormBase ParaSite). This enabled us to identify proteins with closely related homologues in all 3 *Trichuris* species, and we used a statistical test, bootstrap, to assess the confidence scoring of our branches.
- This approach utilises freely available data on the predicted sequences of WAP and CAP gene products as well as previously published data on the protein contents of *T*. *muris* and *T*. *suis* secretions. Including the latter information in our analysis not only gives confidence that these predicted gene products do result in translated proteins, but also is a helpful tool for selecting proteins for further study, since E/S has formed the basis of most vaccination studies thus far and is highly immunogenic.
- We also used the computational tool, InterPro, to predict the number of WAP or CAP domains predicted for each gene product. This analysis was particularly interesting for the WAP protein family as it highlighted the variation in the number of WAP domains between different members, with most encoding 1–3 WAP domains, while some members having up to 15 WAP domains.
- The major disadvantage of this approach was that some of the sequences were not available (e.g., *Tm-*WAP-49), meaning that these gene products could not be included on the phylogenetic trees. There were also some incorrect annotations, i.e., gene products that were initially annotated as WAP or CAP proteins that did not appear to contain these domains upon further structural analysis.
- Furthermore, the genome and transcriptome analyses for *T*. *suis* was carried out by a separate group to the analyses for *T*. *muris* and *T*. *trichiura*, meaning that minor differences in methodology could result in differences in predicted sequences, which could impact on the predicted relatedness of these sequences. However, the *T*. *suis* WAP and CAP proteins were interspersed among those of *T*. *muris* and *T*. *trichiura*, suggesting that any differences in methodology between the Jex and colleagues (2014) and Foth and colleagues (2014) studies did not have a substantial impact.
- Finally, our approach will not categorically identify proteins that will make effective vaccine candidates. Further testing of any of the proteins highlighted in our study in both vaccination studies and in vitro testing to ascertain biological function is necessary. The study does, however, give researchers a starting point on which proteins to prioritise for testing.

## Supporting information

S1 Table List of proteins identified within *T*. *muris* and *T*. *suis* E/S that were initially characterised as WAP or CAP proteins. Ten proteins that were originally characterised as WAP or CAP family members did not appear to have WAP or CAP domains according to structural analysis using InterPro. The UniProt ID and accession code for each protein is listed, along with a reference for the studies in which they were identified [18,26].
(XLSX)
